# The comprehensive cohort model in a pilot trial in orthopaedic trauma

**DOI:** 10.1186/1471-2288-11-39

**Published:** 2011-04-05

**Authors:** Rebecca S Kearney, Juul Achten, Nick R Parsons, Matthew L Costa

**Affiliations:** 1 Warwick Orthopaedics, Clinical Sciences Building, University of Warwick, Coventry, CV2 2DX, UK

## Abstract

**Background:**

The primary aim of this study was to provide an estimate of effect size for the functional outcome of operative versus non-operative treatment for patients with an acute rupture of the Achilles tendon using accelerated rehabilitation for both groups of patients. The secondary aim was to assess the use of a comprehensive cohort research design (i.e. a parallel patient-preference group alongside a randomised group) in improving the accuracy of this estimate within an orthopaedic trauma setting.

**Methods:**

Pragmatic randomised controlled trial and comprehensive cohort study within a level 1 trauma centre. Twenty randomised participants (10 operative and 10 non-operative) and 29 preference participants (3 operative and 26 non-operative). The ge range was 22-72 years and 37 of the 52 patients were men. All participants had an acute rupture of their Achilles tendon and no other injuries. All of the patients in the operative group had a simple end-to-end repair of the tendon with no augmentation. Both groups then followed the same eight-week immediate weight-bearing rehabilitation programme using an off-the-shelf orthotic. The disability rating index (DRI; primary outcome), EQ-5D, Achilles Total Rupture Score and complications were assessed ed at two weeks, six weeks, three months, six months and nine months after initial injury.

**Results:**

At nine months, there was no significant difference in DRI between patients randomised to operative or non-operative management. There was no difference in DRI between the randomised group and the parallel patient preference group. The use of a comprehensive cohort of patients did not provide useful additional information as to the treatment effect size because the majority of patients chose non-operative management.

**Conclusions:**

Recruitment to clinical trials that compare operative and non-operative interventions is notoriously difficult; especially within the trauma setting. Including a parallel patient preference group to create a comprehensive cohort of patients has been suggested as a way of increasing the power of such trials. In our study, the comprehensive cohort model doubled the number of patients involved in the study. However, a strong preference for non-operative treatment meant that the increased number of patients did not significantly increase the ability of the trial to detect a difference between the two interventions.

**Trial registration:**

ISRCTN: ISRCTN29053307

## Background

Randomised controlled trials are accepted as the 'gold standard' in trial design for evaluating the effectiveness of a single intervention, such as a drug[1]. Within the health sector there are a range of non-pharmacological 'complex interventions' that involve an intervention with several interacting components[2]. The practical and methodological difficulties of rigorously evaluating complex interventions are widely documented and have led to the development of alternative approaches to the standard randomised controlled trial[1]. The comprehensive cohort design, where all patients fulfilling the eligibility criteria for a trial can be recruited regardless of their consent to randomisation, is one of those approaches.

This study design has been successfully implemented within musculoskeletal elective settings [3]. However, it has not been evaluated within a trauma setting. Within the context of clinical trials this is a challenging area requiring a dedicated research team to be available as and when patients arrive. Patients are frequently in pain and admitted into an unfamiliar acute care setting with limited time in which to make decisions. This combination of factors makes the process of recruitment for such clinical trials problematic.

The management of Achilles tendon rupture is one such complex intervention. It is a serious and disabling condition affecting approximately 18 per 100,000 people each year, based on data from Scandinavian countries[4,5]. It is associated with prolonged periods off work and much longer abstinence from sporting activity. The injury can be managed operatively (percutaneous or open) or non-operatively (cast immobilization or functional bracing). A Cochrane review of treatment for Achilles tendon ruptureconcluded that patients managed with functional bracing, rather than cast immobilisation, showed a trend towards fewer complications and quicker return to sporting activities[6,7]. There have been several reports which have compared operative and non-operative treatment,[8,9] and the most recent trial used accelerated rehabilitation albeit delayed by two weeks[10]. However, there have been no randomised controlled trials comparing operative with non-operative management where both groups have hadweight bearing mobilisation from the first day of treatment.

Our primary aim was to provide an estimate of possible effect size for the functional outcome of operative versus non-operative treatment of patients with an acute rupture of the Achilles tendon with accelerated rehabilitation for both groups of patients. This would inform the design of subsequent larger trials and enable a sample size analysis to be performed. The secondary aim was to assess the use of a comprehensive cohort research design (i.e. a parallel patient-preference group alongside a randomised group) in improving the accuracy of this estimate within an orthopaedic trauma setting.

## Methods

This study was funded by the British Orthopaedic Foundation (Joint Action) with the kind support of the Rosetrees Trust. Funding was provided to support data collection in the randomised group of patients, but was not available for the inclusion of an additional patient-preference group. Ethical approval for the trial was obtained from Oxfordshire Research Ethics Committee A. The patient-preference data was collected as part of an ongoing service evaluation in this area.

### Participants

Patients were recruited from a fracture clinic within a University Hospital. All patients gave informed consent before taking part in the study. Patients were eligible if they presented with an acute rupture of the Achilles tendon (presentation within 10 days of injury) diagnosed through clinical assessment, had no previous history of Achilles tendon rupture and had no contraindications to surgery. All patients were screened for eligibility by the principal investigator. We invited any eligible patients who did not want to take part in the randomised trial because of a strong preference about treatment, to join one of two non-randomised preference arms.

**Figure 1:**
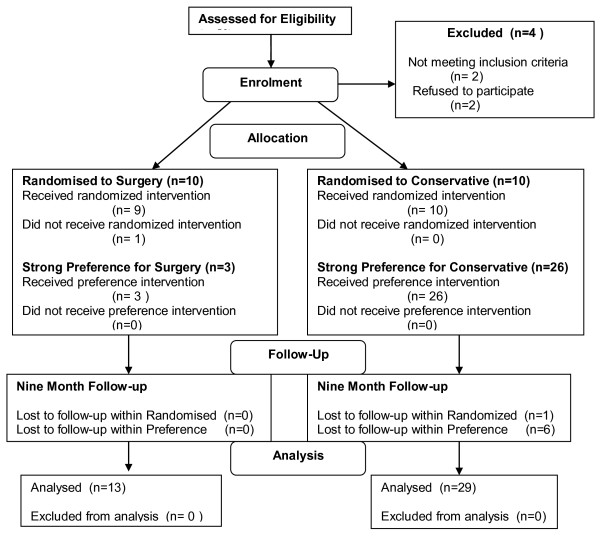
Participant flow after rupture of the Achilles tendon

Recruitment took place betweenAugust 2007 and December 2008. Figure 1 illustrates the flow of participants through the trial. Twenty patients were in the randomised arm (10 allocated to surgery and 10 allocated to non-operative management). Twenty-nine patients were in the preference arm (3 chose surgery and 26 chose conservative management). Two patients did not fulfil the inclusion criteria and two declined to take part in either the randomised trial or preference arms. One patient randomised to the operative group decided not to have surgery, but continued to be followed up on an intention to treat basis.

### Interventions

Both randomised and preference operative groups underwent surgical repair of the Achilles tendon on the next available 'trauma' operating list (within 10 days of injury). The details of the repair technique were recorded but left to the preference of the surgeon. Both operative and non-operative groups followed identical immediate weight bearing rehabilitation protocols. Three one-centimetre heel raises were used within a walking boot (Donjoy, Guildford, UK). Patients were encouraged to remove the boot and move the ankle joint within the limits of their comfort four times each day. Patients then attended fracture clinic every two weeks for eight weeks. At each visit to clinic, one heel wedge was removed and then the boot was removed at week eight. At eight weeks all patients were referred for physiotherapy. The detail of the physiotherapy treatment was left to the preference of the physiotherapist but followed guidelines that allowed gradual progression of strengthening activities. Participation in high-impact sports training was allowed at six months and return to competitive sports at nine months.

### Outcomes

The primary patient reported functional outcome was the Disability Rating Index at nine months[11]. This is a validated general tool which produces a rating on a 100 point scale; where 0 indicates no disability and 100 an inability to perform any activity. Secondary outcome measures were the EuroQol score (0-1)[12], the Achilles total rupture score (ATRS) (0-100)[13] and complication rates. Outcome measures were recorded by a research physiotherapist within the fracture clinic at baseline, six weeks, three months, six months and nine months.

One-hundred percent of outcomes were collected for the randomised groups at baseline, 6 weeks and three months. One patient in the randomised non-operative arm was lost to follow-up at 6 and 9 months. Within the preference arms 100% of outcomes were collected at baseline, 97% at 6 weeks (28/29), 86% at 3 months and 6 months (25/29) and 79% at 9 months (23/29).

### Sample Size

This was a pilot study and a sample size of ten patients in each of the randomised treatment arms was deemed to be sufficient after discussion with clinical experts and the trial statistician, No formal sample size calculation was performed for the pilot but the information from this study will informa power analysis for any larger trial. All of the patients who declined to take part in the randomised trial throughout the recruitment period were offered the option of taking part in the preference study - these patients chose their own treatment but were followed up in the same way as the randomised patients

### Randomisation

A computer-generated 1:1 randomisation sequence was produced and administered by an independent researcher. The treatment allocation was provided by telephone after consent was obtained. The research physiotherapist was not blinded to treatment allocation and it was not possible to blind patients in either group.

### Statistical methods

Observed data for the trial outcome measures were summarised using means and medians as appropriate, depending on the assumed normality or otherwise of each outcome. Variability was assessed using a standard deviation or inter-quartile range. Temporal changes in the relationship between operative and non-operative treatment groups, and associated variability, were demonstrated using box-and-whisker plots. Independent sample t-tests were used to assess the significance of observed differences between operative (surgical) and non-operative (conservative) groups and between randomised and preference groups, for the primary outcome measure (DRI) at nine months post-injury; significance was set at the 5% level. In addition to the main analysis, a multiple regression analysis was undertaken to adjust for the potential confounding effects of patient age and gender prior, to assessing the effects of the main trial treatment factors.

## Results

**Figure 2:**
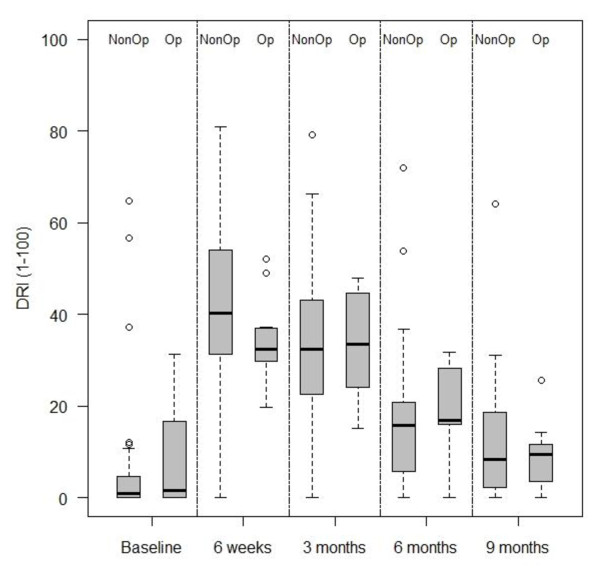
**Box plot of median and inter-quartile ranges for DRI at 9 months for the comprehensive cohort**.

Table 1 shows the clinical and demographic characteristics of the four groups. The baseline characteristics of the randomised and preference, and surgical and non-operative groups were similar. Table 2 shows the mean changes in outcome measures over time, from initial management to follow-up at nine months. Both groups showed gradual improvement over the nine month period. Figure 2 illustrates the median and inter-quartile ranges for the primary outcome measure at the primary outcome point (9 months). These descriptive statistics demonstrate no observable differences between the two groups. Student t-tests and regression analyses demonstrated no evidence of differences between either the operative and non-operative comprehensive cohorts, or between the randomized and non-randomised patients, at the 5% level.

**Table 1:** Baseline demographics and outcome measures

	Surgical		Conservative	
	
	Randomised (n = 10)	Preference (n = 3)	Randomised (n = 10)	Preference (n = 26)
**Age in years**	48 (11)	43 (17)	49 (13)	43 (13)
**Male/Female**	5/5	2/1	8/2	22/4
**Left/Right**	6/4	1/2	6/4	14/12
EQ-5D*	1.0 (0.9-1.0)	1.0 (1.0-1.0)	1.0 (0.8-1.0)	1.0 (1.0-1.0)
ATRS*	100 (92-100)	100 (90-100)	96 (97-100)	100 (90-100)
DRI*	2.6 (0.3-15.4)	0.0 (0.0-10.4)	1.6 (0.3-9.4)	0.4 (0.0-3.2)

ATRS = Achilles Total Rupture Score; DRI = Disability Rating Index. Data are means (SD); *Median and (IQR)

**Table 2:** Changes in outcome measures at six weeks, three, six and nine months.

	Surgical Comprehensive Cohort (n = 13)	Conservative Comprehensive Cohort (n = 36)
**DRI 6 weeks**	34.3 (8.6)	42.27 (19.4)
**DRI 3 Months**	33.5 (11.76	33.7 (16.6)
**DRI 6 Months**	17.5 (10.8)	17.4 (15.9)
**DRI 9 Months**	8.9 (6.9)	12.7 (14.3)

**EQ-5D 6 Weeks**	0.78 (0.17)	0.68 (0.19)
**EQ-5D 3 Months**	0.79 (0.15)	0.75 (0.10)
**EQ-5D 6 Months**	0.85 (0.17)	0.87 (0.14)
**EQ-5D 9 Months**	0.94 (0.11)	0.91 (0.13)

**ATRS 6 Weeks**	41 (21)	35 (18)
**ATRS 3 Months**	39 (13)	39 (17)
**ATRS 6 Months**	64 (23)	62 (25)
**ATRS 9 Months**	78 (21)	76 (21)

Data are means and (SD)

## Discussion

Clinical trials involving surgery are difficult to conduct and especially so in the context of acute injury. The patients are frequently in pain and are admitted into an unfamiliar acute care setting which makes the process of obtaining informed consent problematic. Trials comparing operative and non-operative treatment are particularly difficult as the patient may see the non-operative optionas a 'lesser intervention'. These factors, coupled with the aforementioned difficulties associated with comparing any two complex interventions, have led some authors to question whether randomised controlled trials are possible in this setting[2].

A comprehensive cohort study, where all patients fulfilling the eligibility criteria for a trial are followed up regardless of their consent to randomisation, may be an alternative trial design to overcome these obstacles. Comprehensive cohort designs within the elective setting have demonstrated an 'equal split' within the preference arms [14]. However, because of the increased cost associated with recruiting more patients and collecting more data, and the limited evidence that this investment is beneficial, funding bodies have been reluctant to commit to supporting comprehensive cohorts of patients. This is the first report of a comprehensive cohort study within a trauma setting.

The results of this study have shown that a comprehensive cohort design is acceptable to patients, demonstrated by only 2/51 eligible participants refusing to take part. The high participation rate enables the results to be generalised within the wider context of the NHS. This pilot trial has also shown no clinically relevant or statistically significant differences between operative and non-operatively managed patients, although there is obviously a great potential for type II error [15]. (The standard deviation of the Disability Rating Index in this study was 15 points. Using a more conservative estimate of 20 points for a multi-centre trial and the generally accepted, 'minimum clinically important difference' of 10 points on the DRI, a total sample size of 128 patients would provide 80% power at the 5% level).

Although practically feasible, the benefit of a parallel preference group was undermined in this trauma trial, due to a large majority of patients preferring non-operative management. Consequentially, the estimate of the magnitude of the effect size remains imprecise. Therefore, it has to be questioned whether the increase in staff time and resources required to follow-up the preference group is appropriate.

## Conclusions

Conclusive evidence for the management of acute ruptures of the Achilles tendon is lacking. This is a complex intervention and the Medical Research Council has suggested that the 'traditional' randomised controlled trial design may not be appropriate to evaluate complex interventions. The inclusion of a parallel preference group alongside a randomised controlled trial may increase the sample size, but has cost and resource implications.

Funding bodies appear reluctant to provide the extra cost associated with a comprehensive cohort study design. Inclusion of the parallel preference group increased the number of patients in this trauma study. However, this did not increase the accuracy of the estimate of the treatment effect because of the strong patient preference for one treatment arm.

## Competing interests

The authors declare that they have no competing interests.

## Authors' contributions

RSK was involved in the acquisition, analysis and interpretation of data, drafting and revising the article and final approval. JA was involved in the conception and design, critically evaluating the article and final approval. NRP was involved in the analysis and interpretation, critically evaluating the article and final approval. MLC was involved in the conception and design, interpretation of data, drafting and evaluating the article and final approval.

All authors have read and approved the submission

## Pre-publication history

The pre-publication history for this paper can be accessed here:

http://www.biomedcentral.com/1471-2288/11/39/prepub
